# Decoding the Bioluminescent and Non-Bioluminescent Traits of *Panellus stipticus*: A Genomic and Phenotypic Perspective

**DOI:** 10.3390/jof11110774

**Published:** 2025-10-27

**Authors:** Roel C. Rabara, Xianfa Xie

**Affiliations:** Department of Biology, Center for Biotechnology, Genomics, and Bioinformatics, Virginia State University, Petersburg, VA 23806, USA; roel.rabara@vcuhealth.org

**Keywords:** fungal bioluminescence, *Panellus stipticus*, medium effects, phylogenetic analysis, genomic comparison, luciferase, hispidin synthase, hispidin-3-hydroxylase

## Abstract

The species *Panellus stipticus* presents a unique situation whereby some geographic strains are bioluminescent while others are not. This study investigates the factors affecting the bioluminescence of *P. stipticus*, focusing on culture media optimization, oxygen dependency, and genetic variation between luminescent and non-luminescent strains. Experiments revealed that 10% breadcrumb agar (BCA) significantly enhanced bioluminescence and colony size while supplementation with activated charcoal reduced luminescence. Comparative analysis of carbohydrate-based media showed that BCA outperformed malt extract and molasses in promoting luminescence. Oxygen was confirmed as essential for bioluminescence, with light emission ceasing rapidly under anaerobic conditions and recovering within minutes upon re-exposure to air. Phylogenetic analysis using ITS sequences distinguished luminescent and non-luminescent strains, aligning with biogeographical patterns. Dot plot synteny analysis of draft genomes of a bioluminescent (Panst LUM) and a non-bioluminescent strain (KUC8834) revealed high genomic conservation. However, absence of key bioluminescence genes in non-luminescent strains explains their lack of light emission. Protein sequence comparisons of core enzymes—LUZ, HISP, and H3H—showed functional similarity with Mycenoid lineage species. These findings deepen our understanding of fungal bioluminescence and its genetic and environmental determinants.

## 1. Introduction

Bioluminescence, the biochemical production of light by living organisms, is a fascinating and widespread phenomenon observed across the tree of life. It occurs in diverse groups, including marine bacteria, dinoflagellates, copepods, shrimp, terrestrial fungi, earthworms, and fireflies [1]. This natural light typically results from the enzymatic action of luciferase on the substrate luciferin, in the presence of molecular oxygen. Besides luciferin, other bioluminescent systems, like photoproteins, also exist in nature [1].

In fungi, bioluminescence is restricted to saprotrophic, mushroom-forming species within the Agaricales order and Basidiomycota division. Currently, there are 132 taxa representing five distinct lineages: the *Omphalotus* lineage (Omphalotaceae—18 species), *Armillaria* lineage (Physalacriaceae—14 species), Mycenoid lineage (Mycenaceae—96 species), *Lucentipes* lineage (Cyphellaceae/Porotheleaceae—3 species) [2,3,4,5], and the recently discovered *Eoscyphella* lineage (Cyphellopsidaceae—1 species) represented by *Eoscyphella luciurceolata*, a Cyphelloid species native to the Atlantic Rainforest in São Paulo, Brazil [6].

The fungal bioluminescence pathway is mediated by a gene cluster encoding three key enzymes: luciferase (*luz*), hispidin synthase (*hisps*), and hispidin 3-hydroxylase (*h3h*) [7,8]. Additionally, caffeoyl pyruvate hydrolase (*cph*) plays a role in recycling oxyluciferin into caffeic acid, the precursor substrate for bioluminescence [7,8].

One notable bioluminescent fungal species, *Panellus stipticus*, is a small lignicolous mushroom commonly found on decayed wood in deciduous forests across Europe and North America [9,10]. Despite morphological similarities among specimens from these regions, only North American isolates exhibit bioluminescence [9,11,12]. In contrast, specimens from New Zealand, Russia, and Japan are non-bioluminescent [13,14]. Within North America, bioluminescent strains are predominantly found in the eastern United States, whereas non-bioluminescent strains are more common in the Pacific Northwest [13,15]. Intriguingly, interbreeding studies suggest that these bioluminescent and non-bioluminescent strains belong to the same species, despite their distinct phenotypic traits [9,13,15].

Even among bioluminescent strains in the eastern United States, certain isolates, such as those from Illinois (RHP2416), Georgia, and New Jersey, lack bioluminescence [15,16]. This complexity highlights the need for further investigation into the geographic and phenotypic patterns of bioluminescence.

To deepen our understanding of bioluminescence and phenotypic variations in *P. stipticus*, this study explores factors influencing bioluminescence in vitro and analyzes the population structure of the species through phylogenetic and in silico genomic analyses. These findings could pave the way for improved culturing methods that harness fungal bioluminescence for innovative applications in genetic engineering and molecular imaging.

## 2. Materials and Methods

### 2.1. Strain and Culture Conditions

A bioluminescent strain of *P. stipticus*, designated as isolate C156135, was obtained from Carolina Biological Supply Company (Catalog #156135, https://www.carolina.com/; designated as carLUM, Burlington, NC, USA, access date 1 June 2025). In contrast, a non-bioluminescent strain, designated TFB4313 and originally collected in Switzerland, was kindly provided by the laboratory of Dr. Karen Hughes at the University of Tennessee.

Mycelial cultures of both isolates were cultivated and maintained on 10% breadcrumb agar (BCA) medium. Plates were incubated at 25 °C in complete darkness using a controlled growth chamber to ensure consistent environmental conditions for mycelial development.

### 2.2. Bioluminescence and Colony Growth of P. stipticus

To evaluate the influence of culture media on the growth dynamics and bioluminescent properties of *P. stipticus*, mycelial isolates were cultivated in either standard Petri dishes or six-well microtiter plates containing a variety of growth media.

Mycelial plugs (~2 mm^2^) were aseptically excised from actively growing C156135 stock cultures and transferred to sterile Petri dishes containing one of the following breadcrumb agar (BCA) formulations:

1. 2.5% BCA;
2. 2.5% BCA supplemented with 0.1% activated charcoal;
3. 10% BCA.

Plates were incubated at 25 °C in complete darkness using a temperature-controlled growth chamber. Mycelial colony expansion (diameter in mm) and bioluminescence intensity were measured at 7, 14, and 21 days post-inoculation.

To further assess media-dependent effects, additional mycelial plugs of comparable size were transferred to six-well plates containing the following nutrient media:

• Standard Malt Agar (SMA): 2% malt extract (Fisher Scientific, Massachusetts, MA, USA), 0.2% yeast extract (Fisher Scientific, Massachusetts, MA, USA), 2% agar (Fisher Scientific, Massachusetts, MA, USA) (as per Nussbaum et al. [17]);
• Low Malt Agar (LMA): 0.4% malt extract, 0.2% yeast extract, 2% agar;
• High Malt Agar (HMA): 5% malt extract, 0.2% yeast extract, 2% agar;
• Molasses Yeast Agar (MSY): 2.5% molasses (Fisher Scientific, Massachusetts, MA, USA), 0.5% yeast extract, 1.5% agar (adapted from Bermudes et al. [18]);
• Breadcrumb Agar (BCA): 10% breadcrumb (Walmart, AR, USA), 2% agar (based on Wassink and Kuwabara [19]).

All cultures were maintained at 25 °C under dark conditions for the duration of the experiment.

Light and bioluminescent images were captured using a UVP DigiDoc-It Imaging System (Analytik Jena, CA, USA) equipped with Canon DSLR camera and 8 mm lens. For visual documentation, images under white light were taken using the following camera settings: ISO 200, aperture f/6.3, and exposure time of 1 s. Bioluminescence images were acquired at ISO 800, aperture f/3.5, with an exposure duration of 8 min to enhance signal detection.

Bioluminescence intensity from image captures was quantitatively analyzed using ImageJ software (version 1.54p) [20]. Integrated density values were calculated to assess relative light emission across media and time points.

### 2.3. Anaerobic Suppression of Bioluminescence

To determine the impact of oxygen depletion on light emission, actively growing plates of *P. stipticus* were sealed within an anaerobic chamber containing a Mitsubishi™ AnaeroPack-Anaero Gas Generator (ThermoFisher Scientific, Massachusetts, MA, USA). To ensure that prolonged incubation of the culture in anaerobic conditions will not affect its viability, we only maintain the culture in anaerobic conditions for 24 h. Bioluminescent output was visually monitored at the time of chamber placement and again after a 24 h incubation period under anaerobic conditions. Immediately after removal from the chamber, cultures were re-evaluated for bioluminescence to assess potential reversibility of light suppression.

### 2.4. Biochemical Assay for Bioluminescence Restoration in Non-Bioluminescent Strain

To determine if bioluminescence can be restored in the non-bioluminescent strain of *P. stipticus* through chemical means, isolates of the non-bioluminescent strain of *P. stipticus* (TFB-4313) were cultured in standard malt liquid media (SML) containing 2% malt extract and 0.2% yeast extract [17]. After 14 days of incubation, the cultures were treated with equal concentration (10, 5, 2.5, and 1 µM) of hispidin (HISP), NADPH, caffeic acid (CA), *p*-coumaric acid (CouA), and cinnamic acid (CinA) with sterile water and 40% DMSO serving as controls. The 40% DMSO is the solvent used to dissolve the chemical compounds used in the assay. Bioluminescence was monitored following the previously described protocol. A drop test was also performed on culture grown in 10% BCA. Similar sets of compounds were used at equal concentrations of 100, 10, 1, and 0.5 µM, with water and 40% DMSO as controls. A droplet of each concentration level was dropped on top of 14 day (14 d) old culture and monitored for bioluminescence as described previously.

### 2.5. Phylogenetic Analysis

Exactly 112 internal transcribed spacer (ITS) sequences of *P. stipticus* isolates and related species were downloaded from National Center for Biotechnology Information (NCBI) databases. The evolutionary history was inferred using the Neighbor-Joining method [21] and the optimal tree was generated. The evolutionary distances were computed using the Maximum Composite Likelihood method [22] and are in the units of the number of base substitutions per site. This analysis involved 112 nucleotide sequences. All ambiguous positions were removed for each sequence pair (pairwise deletion option). The final data set comprises 3030 positions. Evolutionary analyses were conducted in MEGA11 [23]. To assess the reliability and accuracy in the estimated phylogenetic tree, bootstrap was performed following 500 replications.

### 2.6. In Silico Analysis of Genomes of Bioluminescent (Panst LUM) and Non-Bioluminescent (KUC8344) Strains of P. stipticus

Genome sequences of bioluminescent strain, herein designated as Panst LUM, and non-bioluminescent strain, designated as KUC8344, of *P. stipticus* were downloaded from Mycocosm (https://mycocosm.jgi.doe.gov/mycocosm/home, access on 1 June 2025) portal [24]. Search of gene cluster of the three genes (*hisps*, *h3h*, and *luz*) involved in the bioluminescence pathway was performed using BLAST (access on 1 June 2025) search tool [25]. To compare genome between the two strains, syntenic dot plot was constructed using D-GENIES software ver. 1.5.0 [26].

To further investigate the similarity and identity of these three genes, homologous sequences were retrieved from *Mycena citricolor* (*mci*), *M. chlorophos* (*mch*), *Armillaria mellea* (*am*), *A. fuscipes* (*af*), *Omphalotus olearius* (*oo*), *Neonothopanus nambi* (*nm*), and *N. gardneri* (*ng*) through BLAST search in the NCBI database using *N. nambi* protein sequences as query. Sequence alignment and phylogenetic construction were performed as described above. Briefly, protein sequences were aligned using standard multiple sequence alignment tools, and percent identity and similarity were calculated to assess sequence conservation. Phylogenetic trees were constructed using the Maximum Likelihood method with bootstrap support to evaluate clustering patterns.

## 3. Results

### 3.1. Effect of Culture Media on Bioluminescence and Colony Growth of P. stipticus

**Figure 1:**
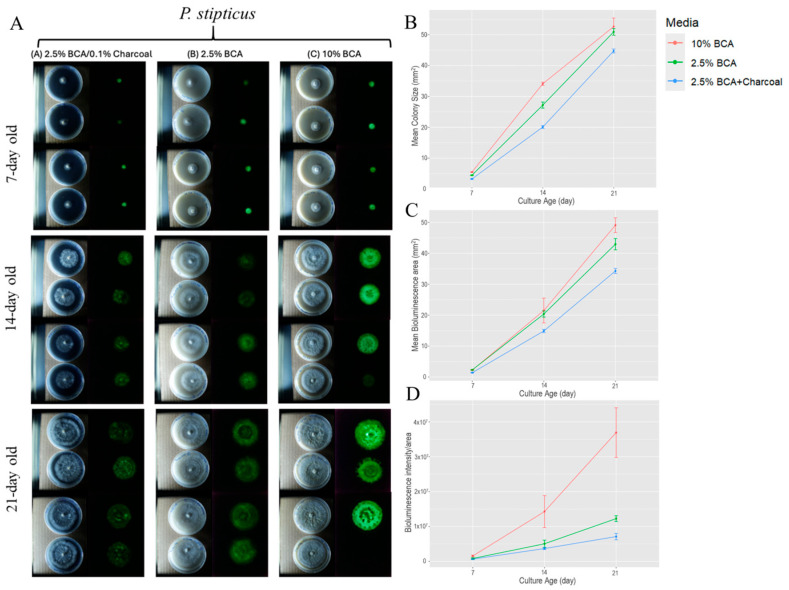
Colony size and bioluminescence area and intensity/area of *P. stipticus* carLUM at 7, 14, and 21 days of culture under various type of media: (**A**) Growth of colony and bioluminescence of *P. stipticus* in 2.5% breadcrumb agar (BCA) with 0.1% charcoal, 2.5% and 10% BCA media; Comparison of colony size (**B**), bioluminescent area (**C**), and bioluminescent intensity/area (**D**) of *P. stipticus* culture at 7, 14, and 21 days of culture under various type of media. Values are mean ± standard error of the mean (SEM) from four replicates.

Figure 1 shows the colony growth and bioluminescence of *P. stipticus* C156135 isolate. Data show that among the three culture media tested, C156135 culture performed well under 10% BCA, followed by 2.5% BCA and 2.5% BCA, with charcoal based on three culture traits of colony size, bioluminescent area, and bioluminescent intensity at different ages of culture. The addition of charcoal in 2.5% BCA resulted in a reduction in bioluminescence intensity of 22%, 27%, and 42% at 7, 14, and 21 days of culture. Colony size was also shown to be affected by charcoal in media composition. Supplementing 2.5% BCA with charcoal resulted in the reduction in colony size of 27%, 26%, and 12% at 7, 14, and 21 days of culture. While the reduction in bioluminescence due to supplementation of charcoal on 2.5% BCA media increases through age of culture, the trend observed in the reduction effect of charcoal on colony size diminished as the culture aged. Comparing the bioluminescent area between 2.5% BCA and one with charcoal supplement showed that the bioluminescent area in 2.5% BCA is 39% higher in 7 day old culture compared to cultures supplemented with charcoal. Then, it dropped down to 20% in 21 day old culture. This trend is similar to what we observed in colony size when comparing the cultures between these two media.

On the other hand, increasing breadcrumb composition four-fold from 2.5% to 10% resulted in an increase in the three traits we measured. Bioluminescence intensity increased by 46, 65, and 67% at 7, 14, and 21 days of culture. Colony size is higher by 20% in 14 d old culture, but a much lower increase (3%) was observed in 21 d old culture. Bioluminescent areas of cultures grown in 10% BCA also increased through time with the highest increase of 12% observed in 21 d old cultures when compared to 2.5% BCA-grown cultures. Statistical analysis revealed that both media and age of culture (day) as well as the interaction of the two factors significantly affect the colony size, bioluminescent area, and bioluminescent intensity of a culture (Tables S1–S3).

**Figure 2:**
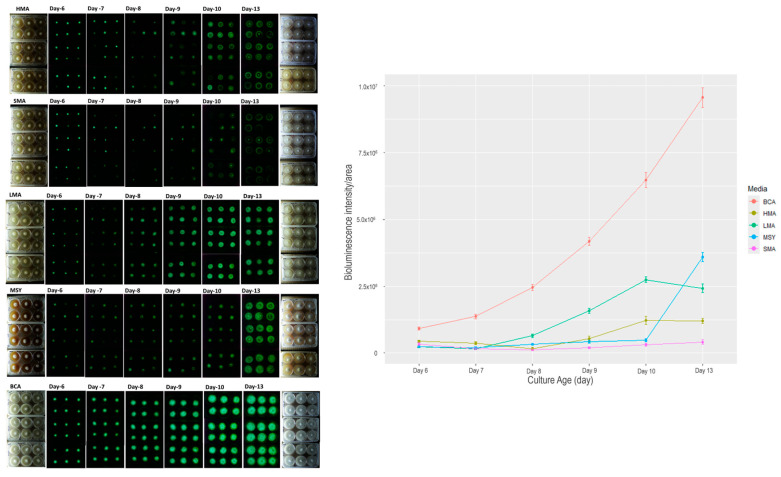
Bioluminescence of *P. stipticus* carLUM strain at 6, 7, 8, 9, 10, and 13 days of culture with various types of media. Values are mean ± SEM bioluminescence intensity/area from the replicates.

One key component in culture media is the energy source to sustain the growth of fungi while in culture. We tested three carbohydrate sources for growth of fungi *P. stipticus* C156135 isolate in vitro: malt extract (MA), molasses (MSY), and bread crumb (BCA) in six-well plates. For malt extract-based media, we tested three malt extract compositions: 0.4% (LMA), 2% (SMA), and 5% (HMA). Among the five media we tested, the culture in BCA showed the highest bioluminescence at each growth stage we measured (Figure 2). Comparison of bioluminescence of culture grown in BCA media and the other four media showed an increase in difference of as much as 96% when BCA is compared to SMA in 13 d old culture. Statistical analysis showed that both media and culture age played significant role in bioluminescence (Table S4).

The effect of malt extract composition can be clearly described in the comparison of bioluminescence in media LMA, SMA, and HMA (Figure 2). Increasing malt extract five-fold from 0.4% (LMA) to 2% (SMA) resulted in a 30% increase in bioluminescence in 6 d old culture. However, in the succeeding days of measurement, the trend is reversed, with LMA showing much higher bioluminescence by 6, 82, 87, 89, and 83% in 7, 8, 9, 10, and 13 d-old cultures. A similar trend was observed in the comparison between LMA and HMA. At an early stage of culture, increasing malt extract by 13-fold led to an increase in bioluminescence of 47 and 54% in 6 and 7 d old cultures. After 7 days, LMA had higher bioluminescence compared to HMA by as much as 74, 66, 55, and 50% in 8, 9, 10, and 13 d old cultures.

### 3.2. Bioluminescence of P. stipticus Under Anaerobic Condition

**Figure 3:**
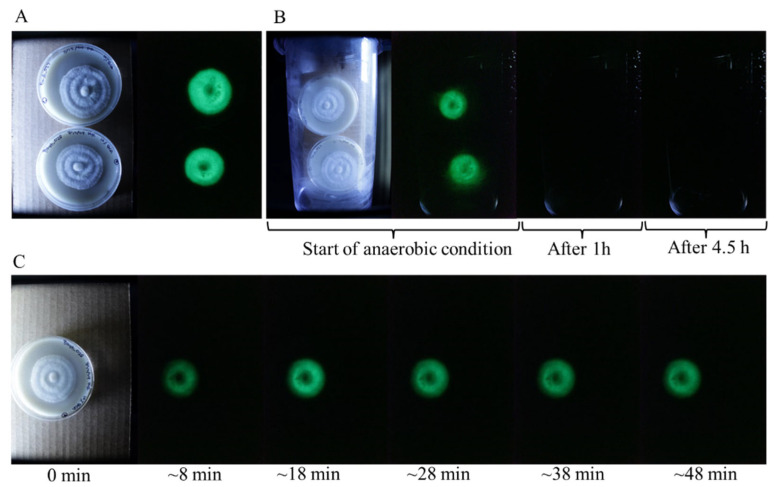
Bioluminescence of *P. stipticus* under anaerobic condition. (**A**) Bioluminescence of isolates prior to subjection to anaerobic condition. (**B**) Culture plates inside the anaerobic chamber. (**C**) Culture plate after removal from the anaerobic chamber.

Molecular oxygen is an essential component of the fungal bioluminescent reaction, serving as a key reactant in the enzymatic oxidation processes that generate light. To investigate the impact of oxygen deprivation on luminescence, we subjected *P. stipticus* C156135 cultures to anaerobic conditions and monitored changes in their bioluminescent activity. Cultures were placed inside a sealed anaerobic chamber, effectively eliminating atmospheric oxygen, and observed over time. Within approximately one hour of exposure to the oxygen-deprived environment, all detectable bioluminescence was completely extinguished, indicating that oxygen is indispensable for sustaining light emission (Figure 3).

To further assess the reversibility of this effect, the cultures were removed from the anaerobic chamber and exposed to ambient air under normal atmospheric conditions. Remarkably, bioluminescence began to recover just eight minutes after reintroduction to an oxygen-rich environment. This rapid restoration of luminescence suggests that the bioluminescence pathway remains intact even after prolonged oxygen deprivation and can resume function as soon as molecular oxygen is available. These findings reinforce the critical role of oxygen in fungal bioluminescence and highlight the sensitivity of the luminescence reaction to environmental oxygen levels.

### 3.3. Biochemical Assay for Bioluminescence Restoration in Non-Bioluminescent Strain

**Figure 4:**
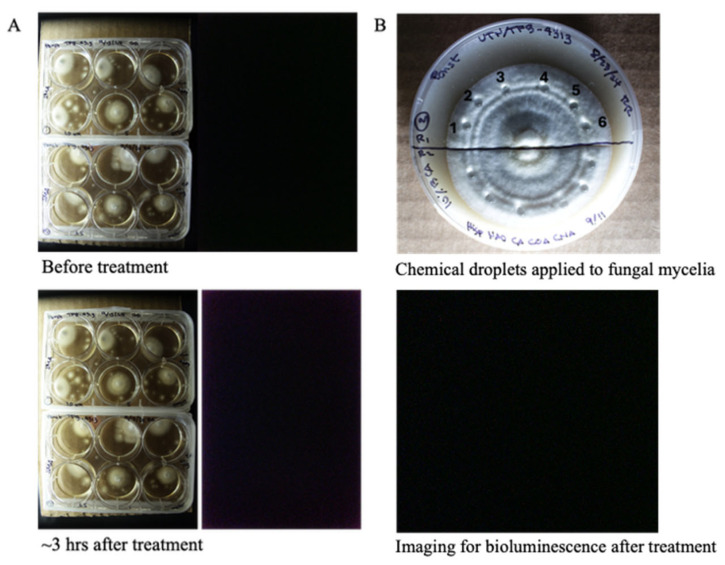
Exogenous application of hispidin, NADPH, caffeic acid (CA), *p*-coumaric acid (CouA), and cinnamic acid (CinA) in non-bioluminescent strain (TFB4313) into cultures grown in liquid media (**A**) and cultures grown in solid media (**B**). Layout of the experiment: 1 = 100 µM, 2 = 10 µM, 3 = 1 µM, 4 = 0.5 µM, 5 = 40% DMSO, 6 = sterile water. Set up is replicated twice (upper and lower half of the plate; chemicals applied as droplets as shown in the plates).

Caffeic acid serves as the primary precursor in the fungal bioluminescence pathway, playing a critical role in the biosynthesis of luminescent compounds. To determine whether bioluminescence could be reactivated in the non-bioluminescent strain *P. stipticus* TFB4313, we supplemented the culture media with the essential substrates and cofactors required for the bioluminescence reaction. Specifically, we introduced equal concentrations of hispidin, NADH, caffeic acid (CA), *p*-coumaric acid (CouA), and cinnamic acid (CinA) at final concentrations of 10, 5, 2.5, and 1 µM into liquid cultures. The cultures were continuously monitored at 10 min intervals over a 3 h period to detect any signs of luminescence. Despite these targeted treatments, no bioluminescent activity was observed under any condition (Figure 4A).

To further assess whether substrate supplementation could induce luminescence in solid-phase cultures, we applied the same set of substrates and cofactors to 14 day old mycelial cultures grown on solid media. Equal concentrations of these compounds (100, 10, 1, and 0.5 µM) were administered as droplets directly onto the surface of the mycelium (Figure 4B). The treated cultures were observed over a period of three days to detect possible luminescent activity. However, even after extended incubation, none of the tested conditions yielded detectable bioluminescence. These results suggest that the non-bioluminescent phenotype of *P. stipticus* TFB4313 may be due to the absence of enzymes involved in the bioluminescence pathway.

### 3.4. Phylogenetic Analysis of P. stipticus

**Figure 5:**
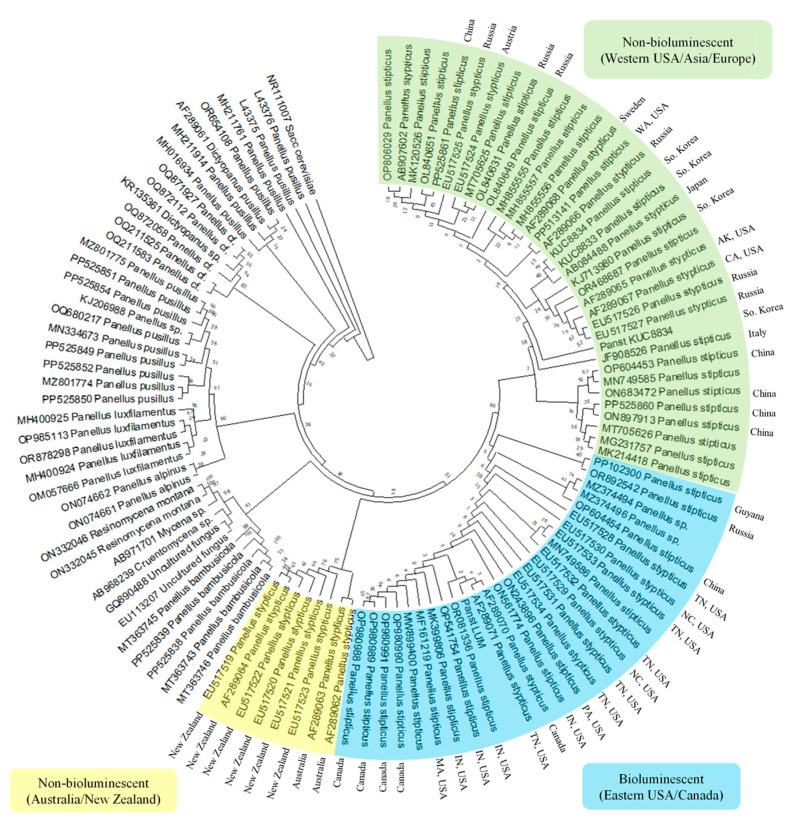
Phylogenetic analysis to infer the evolutionary history of *Panicus stipticus* using internal transcribed spacers (ITS) sequence; inferred using the Neighbor-Joining method [21,22].

The internal transcribed spacers (ITS) region within the eukaryotic ribosomal RNA cistron is widely recognized as the most effective marker for fungal identification and species discrimination (33). In this study, 115 ITS nucleotide sequences from the ribosomal DNA of *P. stipticus* and related fungal species belonging to the genera *Panellus*, *Resinomycena*, and *Mycena* were retrieved from the NCBI database. Phylogenetic analysis grouped *P. stipticus* isolates into three distinct clades (Figure 5).

Group 1 contained isolates from Australia and New Zealand, while Group 2 consisted predominantly of isolates from the eastern United States and Canada. Group 3 included isolates from the western United States, Asia, and Europe. Among these isolates, KUC8834 (from the Korean University collection) and Panst LUM are two *P. stipticus* strains whose genomes have been sequenced, representing non-bioluminescent and bioluminescent variants, respectively. Using KUC8834 as a reference for non-bioluminescent isolates, Group 3 was identified as the non-bioluminescent clade. Conversely, Group 2 was classified as the bioluminescent group based on the sequenced Panst LUM isolate. Interestingly, the bioluminescent Group 2 also included isolates from China (GenBank OP604454), Kaluga Oblast, Russia (GenBank OR892542), and Cuyuni-Mazaruni, Guyana (GenBank PP102300).

Meanwhile, Group 1 formed a distinct clade of non-bioluminescent strains, aligning with previous findings [13], which reported the absence of bioluminescence in *P. stipticus* isolates from New Zealand, Japan, and the former USSR.

### 3.5. Comparative Genomic Analysis of Bioluminescent and Non-Bioluminescent Strains of P. stipticus

To determine whether the two strains of *P. stipticus* (LUM and KUC8344) are similar and belong to the same species, we performed a syntenic dot plot analysis (Figure S1) using genome data obtained from the Mycocosm database. Syntenic dot plots, a type of scatterplot, illustrate putative homologous matches between two sequences and are commonly used for whole-genome comparisons—either within the same genome or across genomes from different taxa—to identify synteny. Synteny refers to two or more genomic regions that originate from a common ancestral genome. Evidence of synteny is established when a set of homologous genes exhibits a collinear arrangement in both genomes. When gene order conservation is observed, the most parsimonious explanation is that these regions share a common evolutionary origin. Syntenic dot plots not only help identify related genomic regions but also highlight genomic areas that have undergone evolutionary changes in one of the genomes being compared.

**Figure 6:**
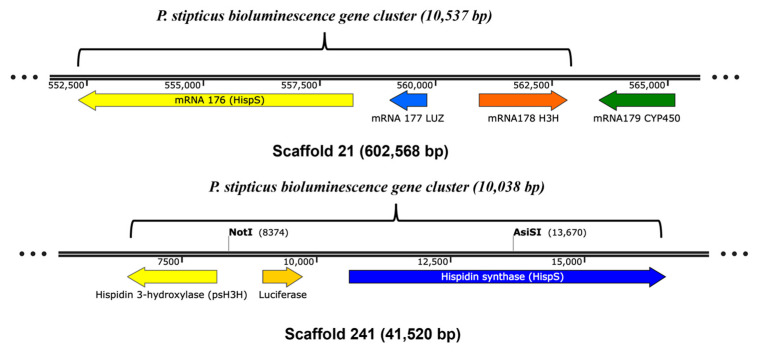
The bioluminescence gene clusters in *P. stipticus* LUM strain genome assembly.

To investigate the presence of genes encoding enzymes involved in the bioluminescence pathway, we performed a BLAST search in the genome of bioluminescent (Panst LUM) and non-bioluminescent (KUC8834) strains for *hisps*, *h3h*, *luz*, and *cph*. The first three genes were shown to be present in two scaffolds: 21 and 241 were in the bioluminescent strain’s genome, which indicates that the sample that was sequenced was a diploid. The locations of these genes within scaffolds 21 and 241 are shown in Figure 6, where they form clusters of approximately 10.5 kb and 10 kb in length, respectively. However, BLAST search of the *cph* gene yielded no result indicating the absence of the gene in the genome assembly for the bioluminescent strain (Panst LUM). Similar BLAST searches of the four genes in the non-bioluminescent strain genome (KUC8834) yielded no hit, indicating the absence of all these genes.

**Figure 7:**
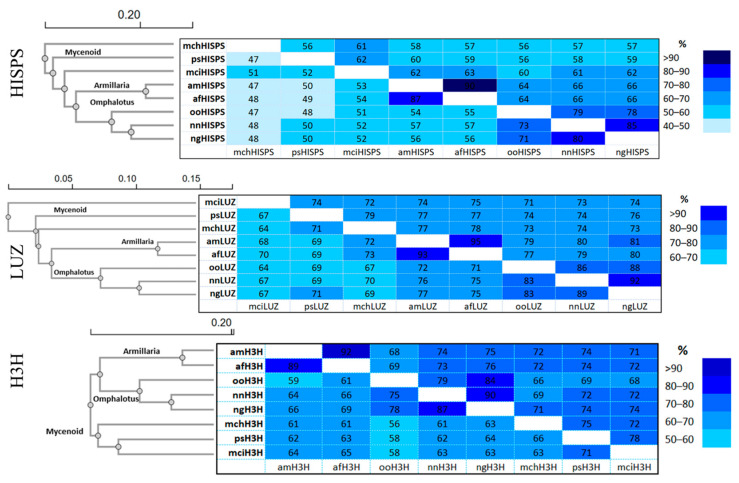
Amino acid sequence identity and similarity matrices of bioluminescence pathway genes in the species of the *Omphalotus*, *Armillaria*, and Mycenoid lineages. Data on top right are the percent similarity and the bottom left are the percent identity. The species used in the analysis are *M. citricolor* (mci), *M. chlorophos* (mch), *P. stipticus* (ps), *A. mellea* (am), *A. fuscipes* (af), *Omphalotus olearius* (oo), *N. nambi* (nm), and *N. gardneri* (ng). Genes that encode enzymes involved in the bioluminescence pathway are *hispidin synthase* (*hisps*), *luciferase* (*luz*), and *hispidin 3-hydroxylase* (*h3h*).

Further phylogenetic, identity, and protein sequence similarity analyses of these three genes in *P. stipticus* were conducted in comparison with homologous sequences from *Mycena citricolor* (mci), *M. chlorophos* (mch), *Armillaria mellea* (am), *A. fuscipes* (af), *Omphalotus olearius* (oo), *Neonothopanus nambi* (nm), and *N. gardneri* (ng), as presented in Figure 7.

Phylogenetic analysis showed that *P. stipticus* clustered with *M. citricolor* and *M. chlorophos* as expected since these three species belong to the Mycenaceae family. Meanwhile, *A. mellea* and *A. fuscipes* clustered together, and both species belong to Physalacriaceae family. On the other hand, members of the Omphalotaceae family, like *O. olearius*, *N. nambi*, and *N. gardneri* clustered together in all three of these proteins.

In phylogenetics, comparing DNA or protein sequences helps measure how alike they are. Percent identity shows exact matches in the sequence, while percent similarity includes gaps and mismatches, using a more detailed scoring system [27]. Identity analysis showed that psHISPS protein is 52% identical to mciHISPS and only 47% identical to mchHISPS. For LUZ protein, psLUZ is 67% identical to mciLUZ and 71% identical to mchLUZ. Meanwhile, psH3H is 73% identical to mciH3H and only 66% identical to mchH3H. Meanwhile, similarity analysis of the HISPS protein revealed that psHISPS shares 62% sequence similarity with mciHISPS, compared to 56% similarity with mchHISPS. Additionally, psLUZ exhibits 74% and 79% similarity to mciLUZ and mchLUZ, respectively. Meanwhile, psH3H shows 78% similarity to mciH3H and 75% similarity to mchH3H. Overall, based on the sequence analysis of the three proteins involved in the bioluminescence pathway, *P. stipticus* is more closely related to *M. citricolor* than to *M. chlorophos*.

## 4. Discussion

The bioluminescence in *P. stipticus* was first described by Atkinson in 1901 and the species and its bioluminescence were systematically described by Buller [11]. Culture media is crucial for studying fungal bioluminescence because it directly influences mycelial growth and luminescence intensity [28]. Optimizing the culture medium allows for maximizing the light-emitting potential of bioluminescent fungi, which is essential for research and our understanding of the phenomenon of bioluminescence in fungi. In order to understand the effect of increasing concentration of the carbohydrate source, we first screened three types of BCA media (2.5% BCA, 2.5% BCA with charcoal, and 10% BCA) using a commercially available strain of bioluminescent *P. stipticus* (C156135 isolate). Our data revealed that bioluminescence of the C156135 isolate is higher in 10% BCA than the other two BCA media. A four-fold increase in bread crumb from 2.5% to 10% led to an increase in bioluminescence of 46% in 7 d old culture and as much as 67% in 21 d old cultures. This also led to an increase in colony size of as much as 20%. The use of bread crumb as substrate for bioluminescent fungi culture was demonstrated as early as 1966 in the culture of *Omphalia flavida* [19]. It has also been used in agar media for maintenance of *P. stipticus* cultures [16,29]. Bread crumb is composed of mostly 77% carbohydrates and showed to be an alternative substrate in *Trametes versicolor* and *Schizophyllum commune* cultivation [30].

To enhance the contrast of growing mycelia in the media, we supplemented our media with activated charcoal. It has been reported that supplementation of charcoal in the media improved the visibility of fluorescence by attenuating approximately 30% of light scattering in aflatoxin-derived fluorescence [31]. When we supplemented 2.5% BCA with 0.1% activated charcoal, it led to a reduction in bioluminescence of 22% in 7 d old culture and as much as 42% in 21 d-old cultures. The reducing effect of charcoal on fungal growth was also reported by Ascough [32] which showed that the addition of charcoal decreases hyphal extension rate in saprophytic fungi *Pleurotus pulmonarius* and *Coriolus versicolor*. We hypothesize that while the addition of charcoal increases the contrast for imaging, it may also absorb some nutrients important for fungal growth, thus reducing the bioluminescence. However, this hypothesis needs to be fully tested in new experiments.

Screening for the three carbohydrate-based media, malt extract (MA), molasses (MSY), and breadcrumb (BCA), showed that BCA media cultures resulted in 75% higher bioluminescence (when compared to LMA and MSY) at early stage of culture (6 d) and as much as 96% higher (compared to SMA) at later stage (13 d) of culture. Malt extract-based media had been used for mycelium cultivation and have shown that higher concentration improved mycelia radial extension and density [17]. Although we did not measure the mycelial density of the cultures from different media, visually it showed that mycelia are denser in BCA compared to the rest of the media.

Bioluminescence in *P. stipticus* is highly dependent on molecular oxygen, like in any bioluminescence, as oxygen plays a fundamental role in the enzymatic oxidation processes that generate light [8]. In this experiment, we sought to understand how the absence of oxygen influences this mechanism by placing cultures of the *P. stipticus* C156135 isolate in an anaerobic chamber.

Observations revealed that luminescence diminished rapidly, fading entirely within an hour of exposure to the anaerobic environment. Given that prolonged oxygen deficiency could also lead to cell death, we limited the anaerobic treatment to maximally 24 h, after which the cultures were able to recover bioluminescence immediately. This suggests that the effect of temporary oxygen deprivation has been only inhibitory on bioluminescence and did not cause any permanent damage to the mycelia. Buller [11] even described that fruiting bodies of *P. stipticus* exposed to carbon dioxide, hydrogen, or nitrogen ceased to emit light in about three seconds and became luminous again in about a second when the fruiting bodies were exposed again to ambient air. All these results suggest that oxygen is not only necessary for sustaining bioluminescence but also that the biochemical reactions involved cease almost immediately upon oxygen deprivation.

Interestingly, when cultures were reintroduced to ambient air, bioluminescence was restored within approximately eight minutes. The luminescence was captured by the camera at 8 min exposure time, so it is possible that the bioluminescence was restored at even shorter than eight minutes. This rapid recovery implies that the enzymatic processes responsible for light emission resume quickly once oxygen is available. The delay between exposure and reactivation may reflect the time required for oxygen diffusion and re-engagement with the necessary luminescence pathways.

These findings reinforce the central role of molecular oxygen in fungal bioluminescence, highlighting its direct influence on the luminescent mechanism of *P. stipticus* and suggesting that bioluminescent activity can be swiftly toggled by modulating oxygen availability.

The existence of both bioluminescent and non-bioluminescent strains of *P. stipticus* was first noted by Buller [11], who observed that specimens collected from England lacked luminescence, whereas those from Canadian cities such as Montreal, Ottawa, and Toronto, as well as U.S. cities including Ann Arbor and St. Paul, exhibited clear bioluminescent properties. Beyond these phenotypic differences, our phylogenetic analysis reveals that these strains can be distinguished based on their internal transcribed spacer (ITS) sequences. The ITS region has been widely accepted as the universal fungal barcode [33] and is routinely employed in identifying uncharacterized fungal strains [34]. Its utility in uncovering biogeographical patterns has been demonstrated in studies of *P. stipticus* across diverse regions such as North America, Eurasia, Australia, and New Zealand [10], which aligned with their findings from restriction fragment length polymorphism (RFLP) analyses. ITS sequencing has also been pivotal in exploring the phylogenetic relationships within the genus *Panellus* [35].

To further investigate the genetic relationship between luminescent and non-luminescent strains, we compared their publicly available draft genomes—Panst LUM and KUC8834, respectively—using dot plot synteny analysis, a well-established genomic comparison method [36]. The analysis revealed extensive conservation in synteny across scaffolds between the two genomes, suggesting that the strains are closely related at the genomic level. Supporting this, earlier research demonstrated mating compatibility between the strains [12,15,16], further affirming that these geographically distinct isolates belong to the same biological species. However, the presence of three key bioluminescence genes, *hisps*, *h3h*, and *luz*, in the bioluminescent strain’s genome and their absence in the non-bioluminescent strain’s genome may explain the phenotypic divergence in bioluminescence between the two strains. Consistent with the genomic analysis, the results from the biochemical assays for bioluminescence restoration in the non-bioluminescent strain suggest that genes coding for the key enzymes in the bioluminescence pathway were missing in the latter. Additionally, our protein sequence analysis of the three core enzymes involved in the bioluminescence pathway—*LUZ*, *HISP*, and *H3H*—showed a high level of similarity to sequences from species within the Mycenoid lineage, corroborating existing reports that place *P. stipticus* within this clade, but also a significant level of divergence while maintaining similar functions for the enzymes.

## 5. Conclusions

Our study highlights the intricate relationship between culture media composition and bioluminescence in *P. stipticus*. Our findings demonstrate that increasing the concentration of breadcrumb substrate significantly enhances both light emission and mycelial growth, establishing 10% BCA as an optimal medium among those tested. In contrast, the addition of activated charcoal had a suppressive effect on luminescence, likely due to its impact on fungal metabolism and growth dynamics. Comparative screening with other carbohydrate-rich media further confirmed BCA’s superior support for sustained and intense bioluminescence.

The crucial role of oxygen in the light-producing mechanism was underscored by the rapid suppression of luminescence under anaerobic conditions and subsequent recovery in aerobic conditions. This oxygen dependency not only mirrors earlier observations but also opens the door to controlling bioluminescence through environmental manipulation. Moreover, molecular and genomic analyses revealed that bioluminescent and non-bioluminescent strains, while phenotypically distinct, are largely similar across the genomes and belong to the same species, but the presence/absence of key genes involved in the bioluminescence pathway may be the key for the phenotypic divergence.

Altogether, these results deepen our understanding of fungal bioluminescence, underscore the importance of optimizing culture conditions for experimental and potential biotechnological applications, and pave the way for future studies on the molecular regulation, structural, and functional genomics of bioluminescent fungi versus non-luminescent strains/species.

## Data Availability

The data supporting reported results in Figure 5 and Figure 7 can be found in FigShare.com with identifiers 10.6084/m9.figshare.30279622 and 10.6084/m9.figshare.30113593, respectively.

## Supplementary Materials

The following supporting information can be downloaded at https://www.mdpi.com/article/10.3390/jof11110774/s1, Figure S1: Dot plot analysis of *P. stipticus* genomes between bioluminescent and non-bioluminescent strains; Table S1: Two-factor ANOVA on the effect of growth media on bioluminescence intensity of *P. stipticus* culture at 7, 14, and 21 days of culture; Table S2: Two-factor ANOVA on the effect of growth media on colony size (mm^2^) of *P. stipticus* culture at 7, 14, and 21 days of culture; Table S3: Two-factor ANOVA on the effect of growth media on area (mm^2^) of bioluminescence of *P. stipticus* culture at 7, 14, and 21 days of culture; Table S4: Two-factor ANOVA on the effect of growth media on bioluminescence of *P. stipticus* culture at 6, 7, 8, 9, 10, and 13 days of culture.

## Abbreviations

The following abbreviations are used in this manuscript:

luz	luciferase
hisps	Hispidin synthase
h3h	Hispidin 3-hydroxylase
cph	caffeoyl pyruvate hydrolase
BCA	Breadcrumb Agar
SMA	Standard Malt Agar
LMA	Low Malt Agar
HMA	High Malt Agar
MSY	Molasses Yeast Agar
CA	Caffeic Acid
CouA	*p*-coumaric acid
CinA	Cinnamic Acid
NADPH	Nicotinamide Adenine Dinucleotide Phosphate
DMSO	Dimethyl Sulfoxide
ITS	Internal Transcribed Spacer
NCBI	National Center for Biotechnology Information
