# Supplementary material for: Decoding the Bioluminescent and Non-Bioluminescent Traits of Panellus stipticus: A Genomic and Phenotypic Perspective
Source: J Fungi (Basel). 2025 Oct 27;11(11):774. doi: 10.3390/jof11110774 (PMC12653593; doi:10.3390/jof11110774)
Supplement: Supplementary file 1 [file jof-11-00774-s001.zip › jof-3898318-supplementary.pdf]

Supplementary Materials: The following supporting information can be downloaded at: https://www.mdpi.com/article/doi/s1

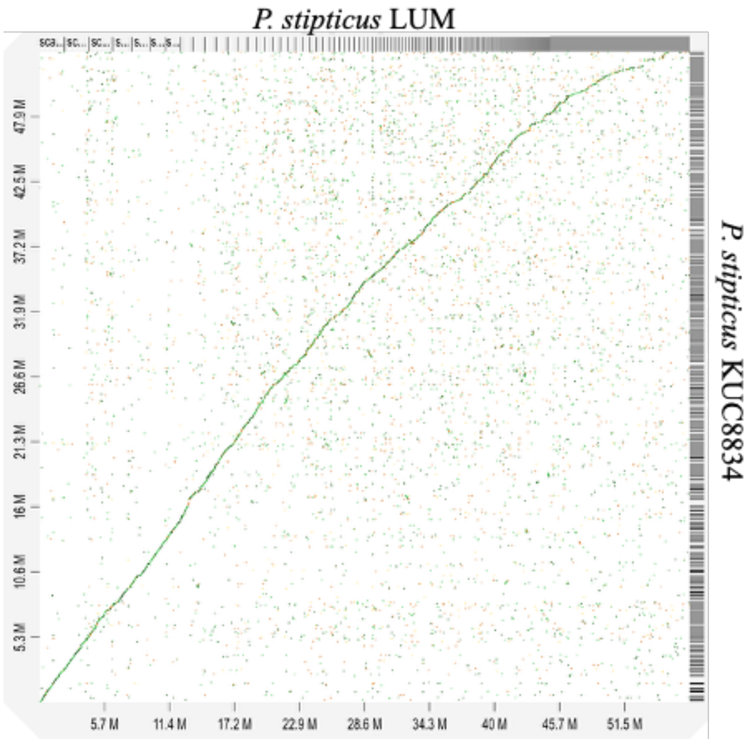

Figure S1. Dot plot analysis of *P. stipticus* genomes between bioluminescent and non-bioluminescent strains.

Table S1: Two-factor ANOVA on the effect of growth media on bioluminescence intensity of *P. stipticus* culture at 7, 14 and 21 days of culture.

Source of Variance	D.f.	Sum Sq	Mean Sq	F-value	Pr(>F)	
Media	2	9.254e+14	4.627e+14	17.443	1.57e-05	***
Day	2	1.631e+15	8.155e+14	30.742	1.41e-07	***
Media x Day	4	8.777e+14	2.194e+14	8.272	0.000192	***
Residuals	26	6.897e+14	2.653e+13			

Level of significance: * p<0.05, ** p<0.01, *** p = <0.001

Table S2: Two-factor ANOVA on the effect of growth media on colony size (mm^2) of *P. stipticus* culture at 7, 14 and 21 days of culture.

Source of Variance	D.f.	Sum Sq	Mean Sq	F-value	Pr(>F)	
Media	2	393	196	41.025	6.54e-09	***
Day	2	12146	6073	1269.186	< 2e-16	***
Media x Day	4	147	37	7.672	0.000289	***
Residuals	27	129	5			

Level of significance: * p<0.05, ** p<0.01, *** p = <0.001

Table S3: Two-factor ANOVA on the effect of growth media on area (mm^2) of bioluminescence of *P. stipticus* culture at 7, 14 and 21 days of culture.

Source of Variance	D.f.	Sum Sq	Mean Sq	F-value	Pr(>F)	
Media	2	204	102	8.755	0.00124	**
Day	2	9102	4551	390.257	< 2e-16	***
Media x Day	4	186	46	3.977	0.01198	*
Residuals	26	303	12			

Level of significance: * p<0.05, ** p<0.01, *** p = <0.001

Table S4: Two-factor ANOVA on the effect of growth media on bioluminescence of *P. stipticus* culture at 6, 7, 8, 9, 10 and 13-day of culture.

Source of Variance	D.f.	Sum Sq	Mean Sq	F-value	Pr(>F)	
Media	4	1.05E+15	2.64E+14	1057.6	<2e-16	***
Day	5	6.43E+14	1.29E+14	516.0	<2e-16	***
Media x Day	20	6.55E+14	3.27E+13	131.4	<2e-16	***
Residuals	510	1.27E+14	2.49E+11			

Level of significance: * p<0.05, ** p = p<0.01, *** p = <0.001
